# Feedback Signaling between Cholangiopathies, Ductular Reaction, and Non-Alcoholic Fatty Liver Disease

**DOI:** 10.3390/cells10082072

**Published:** 2021-08-12

**Authors:** Tianhao Zhou, Debjyoti Kundu, Jonathan Robles-Linares, Vik Meadows, Keisaku Sato, Leonardo Baiocchi, Burcin Ekser, Shannon Glaser, Gianfranco Alpini, Heather Francis, Lindsey Kennedy

**Affiliations:** 1 Division of Gastroenterology and Hepatology, Department of Medicine, Indiana University School of Medicine, Indianapolis, IN 46202, USA; zhouv@iu.edu (T.Z.); debkundu@iu.edu (D.K.); vikmead@iu.edu (V.M.); keisato@iu.edu (K.S.); galpini@iu.edu (G.A.); heafranc@iu.edu (H.F.); 2 Department of Graduate Studies, Indiana University School of Medicine, Indianapolis, IN 46202, USA; jonroble@iu.edu; 3 Liver Unit, Department of Medicine, University of Rome Tor Vergata, 00133 Rome, Italy; baiocchi@uniroma2.it; 4 Division of Transplant Surgery, Department of Surgery, Indiana University, Indianapolis, IN 46202, USA; bekser@iu.edu; 5 Department of Medical Physiology, Texas A&M University College of Medicine Bryan, Bryan, TX 77807, USA; sglaser@tamu.edu; 6 Richard L. Roudebush VA Medical Center, Department of Research, Indianapolis, IN 46202, USA

**Keywords:** cholangiocytes, bile acids, cholestatic liver disease, ductular reaction, non-alcoholic fatty liver disease

## Abstract

Fatty liver diseases, such as non-alcoholic fatty liver disease (NAFLD), are global health disparities, particularly in the United States, as a result of cultural eating habits and lifestyle. Pathological studies on NAFLD have been mostly focused on hepatocytes and other inflammatory cell types; however, the impact of other biliary epithelial cells (i.e., cholangiocytes) in the promotion of NAFLD is growing. This review article will discuss how cholestatic injury and cholangiocyte activity/ductular reaction influence NAFLD progression. Furthermore, this review will provide informative details regarding the fundamental properties of cholangiocytes and bile acid signaling that can influence NAFLD. Lastly, studies relating to the pathogenesis of NAFLD, cholangiopathies, and ductular reaction will be analyzed to help gain insight for potential therapies.

## 1. Cholangiocytes—The Fundamentals

### 1.1. Structure, Function, and Heterogeneity

The liver is a regenerative gland comprised of parenchymal cells (i.e., hepatocytes) and non-parenchymal cells (e.g., cholangiocytes, epithelial cells lining the biliary epithelium). Cholangiocytes play a role in the modification of canalicular bile, but they are also the primary target of damage in cholangiopathies, such as primary sclerosing cholangitis (PSC) and primary biliary cholangitis (PBC) [1]. While cholangiocytes were initially known for their modulation of bile secretion [2], they have become increasingly recognized for their impact on biliary and liver diseases via the release of various autocrine/paracrine neurotransmitters, neuropeptides, and gastrointestinal hormones during chronic cholestatic liver damages [3]. Under normal conditions, cholangiocytes have low proliferative capability, but following injury or specific stimuli, cholangiocytes undergo proliferation and other phenotypic changes [3]. Following injury, cholangiocytes enter a neuroendocrine phenotype and secrete a plethora of neurotransmitters, growth factors, and other mediators, which influence the surrounding microenvironment and modulate biliary damage (autocrine) as well as inflammation and liver fibrosis (paracrine) pathogenesis [2,4]. Recently, our understanding of the impact of activated cholangiocytes on other liver diseases, including non-alcoholic fatty liver disease (NAFLD), has increased.

**Figure 1:**
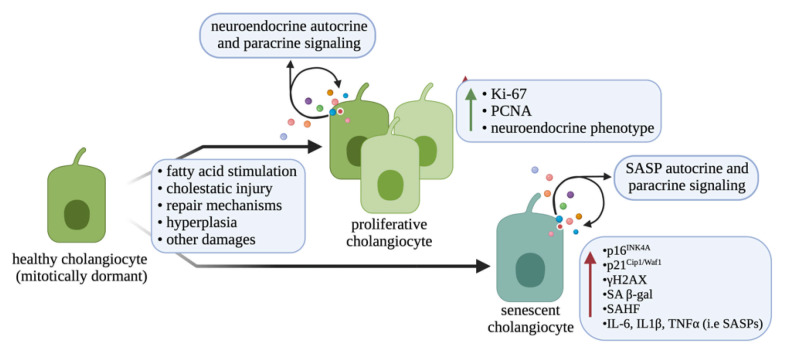
Molecular differences between healthy, proliferative and senescent cholangiocyte. After exposure to various damaging effects, cholangiocytes can undergo changes in either their proliferative response or induction of senescence. Different cellular changes, such as enhanced expression of proliferative markers and correlation with the neuroendocrine phenotype are associated with proliferative cholangiocytes. Following induction of senescence, cholangiocytes can increase their expression of senescent factors, have senescence-associated heterochromatin foci (SAHF), and begin to secrete various senescence-associated secretory phenotype (SASP) factors. Other biliary damages (i.e., apoptosis, autophagy, and others) are not shown. Image made with BioRender (https://biorender.com/).

In humans, cholangiocytes are classified based on their size (small, medium, and large), which corresponds to the diameter of the biliary tract [5]. Differing from humans, rodents present with small and large bile ducts that are lined by small and large cholangiocytes, respectively, having differing functional properties [5]. Small cholangiocytes are thought to be a biliary-committed progenitor cell compartment in the liver, and are more resistant to damage than large cholangiocytes [2,6]. However, large cholangiocytes are considered mature cholangiocytes and can express cyclic adenosine monophosphate (cAMP)-dependent functional markers, such as secretin, secretin receptor (SR), cystic fibrosis transmembrane receptor (CFTR), and anion exchanger 2 (AE2), and are distinct from small cholangiocytes [7]. Secretin binds to basolateral SR, which in turn causes activation of CFTR and subsequent opening of AE2, leading to the release of bicarbonate into the lumen of the bile ducts (i.e., composing the so-called bicarbonate umbrella) [8]. Further, damaged large cholangiocytes can become senescent and enter a senescence-associated secretory phenotype (SASP), whereby they release inflammatory mediators that promote further damage [9]. However, following damage to large ducts, small cholangiocytes undergo proliferation and transdifferentiation to a large cholangiocyte phenotype to aid in replenishing the epithelial lining after injury [6]. Understanding the differential response of small and large cholangiocytes to damage is important considering different liver diseases may affect specific portions of the bile duct, leading to varying biliary responses. The different cellular changes associated with cholangiocyte proliferation or senescence are described in Figure 1.

### 1.2. Cholestasis and Cholangiopathies

Cholestasis is defined as the blockage of bile flow from the liver into the bile duct system, and this obstruction leads to hepatic bile acid accumulation and subsequent damage [10]. Cholestasis can be classified as either intrahepatic or extrahepatic based on location of blockage, and is a hallmark of cholangiopathies, but the impact of cholestasis in other chronic liver diseases is becoming more known. There are several clinical conditions that contribute to the onset of cholestasis, including alcohol-induced liver disease, acute hepatitis, cholangitis, and potentially NAFLD [11]. As previously stated, during normal physiological conditions, cholangiocytes are mitotically quiescent; however, they can become proliferative, proinflammatory, pro-fibrotic, or senescent in response to damage, including cholestasis, and in the context of cholangiopathies, such as PBC, PSC, and cholangiocarcinoma (CCA) [12,13].

PBC is an autoimmune disease affecting the small bile ducts in the liver, in which the cholangiocytes become inflamed and develop scar tissue, causing the bile ducts to become stiff and leading to significant narrowing in the lumen. A common clinical symptom in PBC patients includes dry skin, xanthomas, and pruritus [14], and the diagnosis is usually based on the presence of serum anti-mitochondrial antibodies (AMAs) [15]. PBC diagnosis can overlap with autoimmune hepatitis, potentially owing to both diseases being autoimmune disorders [15]. Currently, ursodeoxycholic acid (UDCA) and/or obeticholic acid (OCA) treatment are the only FDA-approved therapies for PBC [15], and specifically, OCA therapy has shown improved hepatic bile acid excretion in PBC patients [16].

PSC is a chronic liver disease affecting either the small or large bile ducts, causing inflammation, reduced bile flow, and fibrosis [17], and patients with PSC may encounter symptoms such as fatigue, itching, and abdominal pain [18]. Unfortunately, there is currently no effective treatment available for this disease [17], and the pathogenesis of PSC is not completely understood; however, it is believed to be strongly related to intestinal disorders as a large proportion (~90%) of patients with PSC present with inflammatory bowel disease (IBD) [19]. Although PSC is currently considered a rare disease, data suggest that the prevalence of PSC is rising [18].

CCA is a malignant growth developing from cholangiocytes and is classified relative to its location (intrahepatic, perihilar, and distal) [20]. Similar to PSC, CCA is a rare and life-threatening disease that currently has no curative treatment options other than surgery [20]. Data have shown that patients from Western countries with PSC have an increased risk of developing CCA [21].

### 1.3. Ductular Reaction

**Figure 2:**
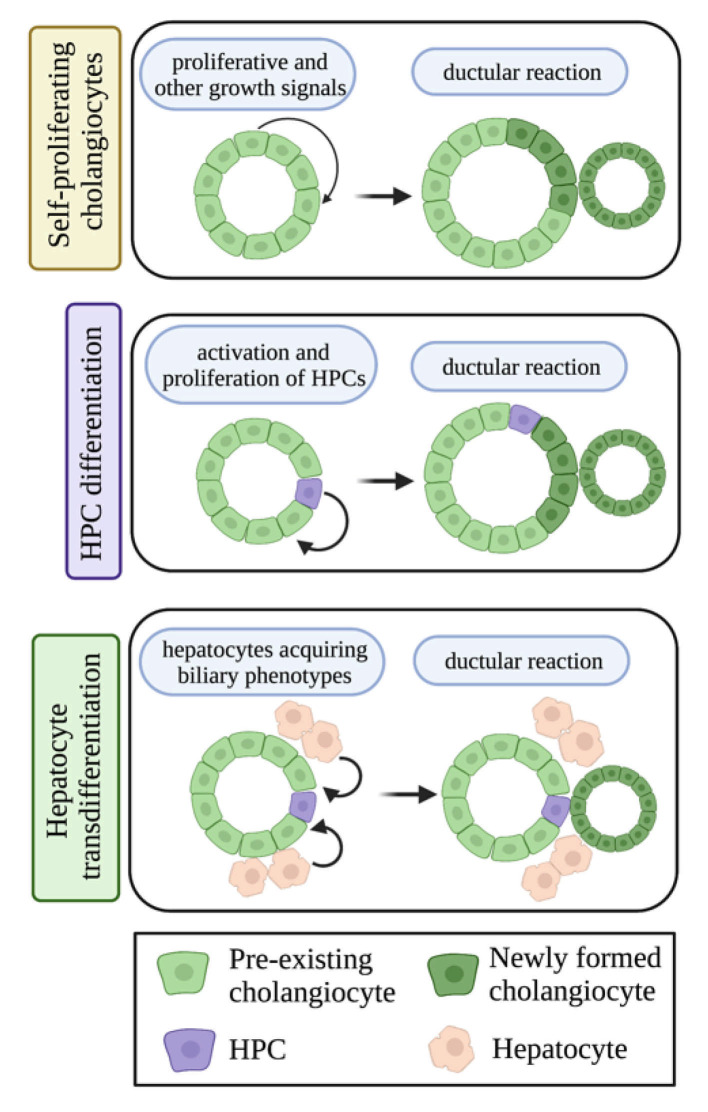
Different cellular compartments can contribute to ductular reaction. Pre-existing cholangiocytes can undergo self-proliferation to generate ductular reactive cholangiocytes; HPCs can become activated, proliferate, and differentiate to a cholangiocyte, thus contributing to ductular reaction; and hepatocytes can acquire biliary phenotypes and undergo transdifferentiation to a cholangiocyte-like cell type and generate ductular reaction. Image made with BioRender (https://biorender.com/).

Ductular reaction is histologically defined as biliary proliferation or hyperplasia [22]. The origin of ductular reaction is highly debated and has been attributed to (i) small cholangiocyte proliferation, (ii) activation and proliferation of hepatic progenitor cells (HPCs), or (iii) the transdifferentiation of hepatocytes to cholangiocytes [3,22]. Ductular reaction has been implicated in cholangiopathies, but is also associated with chronic liver disorders [22]. The HPCs are considered the stem cell niche within the liver and are found at the terminal ends of bile ducts, and it is important to note that HPCs are considered a primordial hepatic stem cell population that can differentiate into multiple hepatic cell types [22]. As previously stated, HPCs are uniquely capable of differentiation into various liver cells, including cholangiocytes, after hepatic injury, and may contribute to ductular reaction [23]. On the other hand, hepatocytes have also been considered as an important source of ductular reaction [22,24,25]. A small number of hepatocytes have been found to express the cholangiocyte-specific marker (SR) in non-alcoholic steatohepatitis (NASH) patients and WT mice fed with a Western diet [26]. However, whether the SR-positive hepatocytes are transdifferentiating to cholangiocytes or not needs to be further investigated. The different compartments of ductular reaction, including self-proliferating cholangiocytes, HPC differentiation and proliferation, and hepatocyte transdifferentiation are described in Figure 2. According to recent research, ductular reaction could be a potential target for liver disease therapies [22], and ductular reaction has been implicated in worsening liver fibrosis [22,27,28]. Further research is needed to confirm that ductular reaction indeed stimulates the development of fibrosis, and its potential role in NAFLD. 

### 1.4. Bile Acid Signaling

Bile acids are catabolized from cholesterol and are considered digestive surfactants for their role in lipid absorption and emulsification [29]. Bile acids play key roles in lipid homeostasis in the liver by activating different kinds of receptors and movement across membranes via various transporters, such as farnesoid X receptor (FXR), transmembrane G-protein coupled receptor (TGR5), and sphingosine-1-phosphate receptor (S1PR)2 [30]. It is widely known that plasma bile acid levels are significantly increased in patients with NASH [31], thus suggesting a possible role of bile acid signaling in NAFLD phenotypes.

Various hepatic and intestinal cells can transport bile acids across their plasma membranes via bile acid transporters, including the organic solute transporters (OSTs), bile salt export pump (BSEP), Na^+^-taurocholate cotransporting polypeptide (NTCP), and multidrug resistant-associated protein 2 (MRP2). During cholestasis, cholangiocytes express and utilize organic solute transporters (OSTα-OSTβ) as a protective mechanism to respond to bile acid accumulation and avoid cytotoxicity [32]. Specifically, OSTα and OSTβ form heterodimers in order to transport bile acids across the cholangiocyte basolateral membrane, lending to their role in the hepatic bile acid reabsorption [33]. NTCP is found only on hepatocytes and mediates their uptake of bile acids from enterohepatic circulation; however, BSEP and MRP2 can be found in the bile canaliculus between hepatocytes and cholangiocytes [34]. Genetic loss of BSEP is linked to the development of progressive familial intrahepatic cholestasis [35], and off-target inhibition of BSEP is associated with cholestatic injury in the setting of drug-induced liver injury [36].

Aside from bile acid transport, various membrane and nuclear receptors modulate downstream bile acid effects. FXR is a nuclear receptor that is considered the master regulator of bile acid synthesis and secretion [37]. Furthermore, FXR can be activated by conjugated and unconjugated hydrophobic bile acids, but not hydrophilic bile acids [38]. Indeed, it is widely known that FXR activation reduces hepatic bile acid synthesis, promotes bile acid secretion into bile, and reduces hepatic bile acid uptake [39,40]. Interestingly, variants in FXR are only responsible for a few cholestatic syndromes [41]. OCA is an agonist of FXR, which has been approved for treating PBC patients. Positron emission tomography (PET) scan showed that OCA significantly increased the transport of bile acids from blood to bile and reduced the exposure time of liver to potentially toxic bile acids [16]. TGR5 is predominantly found on cholangiocyte cilia and is activated by various bile acids to promote cAMP signaling and potential bicarbonate secretion into bile [42]. S1PR2 is another important membrane-bound receptor that mediates bile acid-induced cholestatic liver injury [43] that is activated by conjugated bile acids, but not unconjugated bile acids [44]. In BDL mice, the activation of S1PR2 and down regulation of apical sodium-dependent bile acid transporter (ASBT) promotes cholangiocyte proliferation through the ERK1/2 signaling pathway. BDL-induced cholestatic liver injury was markedly reduced in S1PR2^−/−^ mice. The S1PR2 antagonist, JTE-013, also showed protective effect against BDL-induced cholangiocyte proliferation, suggesting that S1PR2 plays a crucial role in bile acid-induced cholestasis [43].

### 1.5. Biliary Neuroendocrine Phenotype

**Figure 3:**
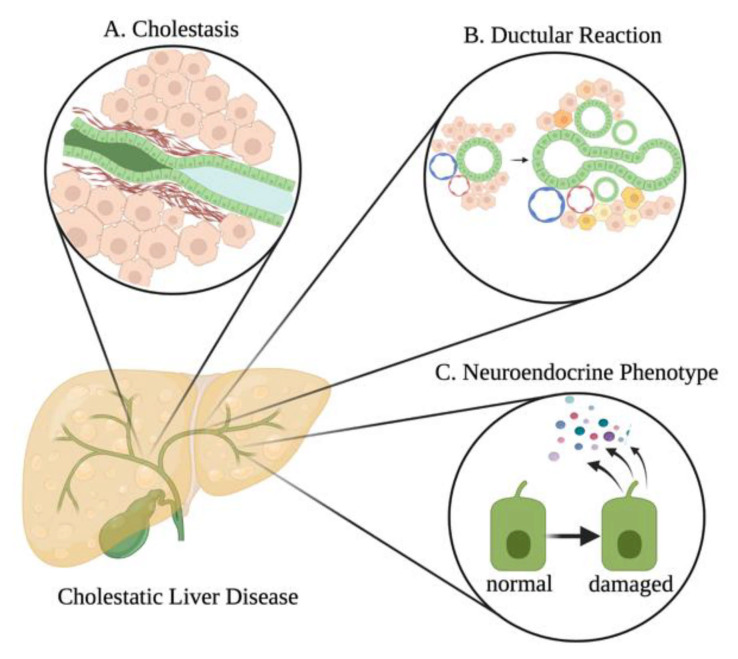
Histopathology of bile duct damage. Different bile duct damages associated with cholestatic liver diseases. (**A**) Following bile duct damage, stricturing of the bile ducts can occur from various processes, including significant peribiliary fibrosis. This stricturing can lead to cholestasis (i.e., blockage of drainage of bile from the liver). (**B**) Cholestatic injury can lead to ductular reaction, which can be accompanied by enhanced biliary inflammation and fibrosis. (**C**) Following damage, cholangiocytes can enter a neuroendocrine phenotype, whereby they increase their synthesis and secretion of various growth factors, neurohormones, cytokines, chemokines, and other factors that can modulate liver damage through autocrine and paracrine signaling mechanisms. Image made with BioRender (https://biorender.com/).

Following damage, cholangiocytes can become activated and enter a neuroendocrine phenotype and begin to release a plethora of factors [45]. Furthermore, these neuroendocrine-like cholangiocytes have increased expression and secretion of serotonin, opioid peptides, hormones, and neuropeptides [46]. Secretin is one of the well-studied hormones involved in cholangiocyte function and proliferation, and its expression and synthesis is enhanced in injured and proliferative cholangiocytes [12]. Secretin stimulates the proliferation of large cholangiocytes by reducing the expression of microRNA (miR)-125b, which in turn increases the expression of vascular endothelial growth factor (VEGF) [47]. In rodents, inhibition of secretin/SR signaling or genetic knockout of secretin or SR decreases biliary hyperplasia and liver fibrosis in cholestatic models [12]. Humans further express secretin/SR that signals in a similar manner to mice. Activated cholangiocytes can also synthesize and respond to histamine that can either stimulate or inhibit cholangiocyte proliferation, depending on the sub-type of histamine receptor (HR) to which histamine binds [48]. Each HR can activate various pathways for cell growth as they interact with unique G proteins [48]. It is known that small cholangiocytes primarily express H1HR, whereas large cholangiocytes express H2HR, and binding of histamine to H1 or H2 HR promotes biliary proliferation and subsequent liver fibrosis [48,49]. It has been largely demonstrated that loss of histamine signaling or inhibition of H1 or H2 HR is able to ameliorate biliary proliferation and subsequent liver fibrosis in models of cholestasis [50,51,52]. Biliary neuroendocrine phenotype induced in cholestasis and other chronic liver diseases is associated with an atypical ductular reaction and is generally associated with pronounced inflammatory responses and peribiliary fibrosis [46], thus understanding this compartment during NAFLD may be key. Different damages associated with cholestatic injury such as NAFLD include cholestasis, ductular reaction, and the biliary neuroendocrine phenotype, which are described in Figure 3.

## 2. Non-Alcoholic Fatty Liver Disease

**Figure 4:**
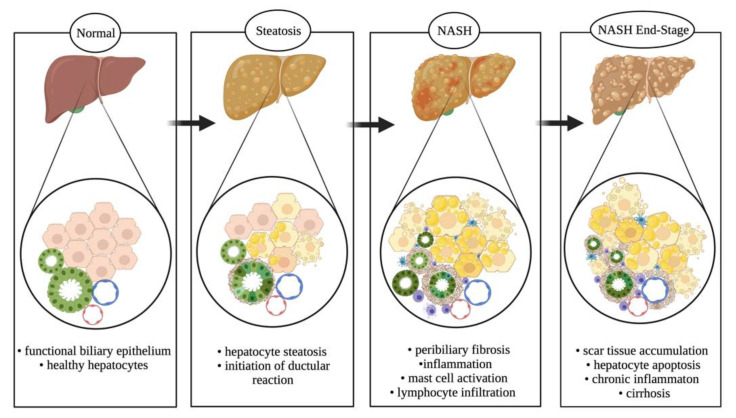
NAFLD spectrum. NAFLD progresses from a healthy liver parenchyma (steatosis < 5% of hepatocytes) to a stage (steatosis > 5% of hepatocytes) with initiation of ductular reaction, mast cells and macrophage infiltration, and peribiliary fibrosis coupled with biliary senescence. It progresses further to a severe stage of NASH with scar tissue accumulation, elevated steatosis and hepatic ballooning, and increased circulating cytokine and SASP secretion from senescent bile ducts. Upon persistent dietary conditions, it assumes a chronic phenotype resulting in hepatocellular carcinoma and cirrhosis. Image made with BioRender (https://biorender.com/).

NAFLD is one of the most common liver disorders, especially in Western countries [53]. NAFLD is characterized by excessive lipid deposition (i.e., steatosis) in the liver and is concerning for developing cirrhosis and liver cancer [54]. Further, there is a strong association with obesity and metabolic syndrome in patients with NAFLD [53]. It is estimated that more than 80 million people in the United States are affected by NAFLD [55]; it should also be noted that children and adolescents are at risk for developing NAFLD, and the prevalence is likely to continue growing [56]. NAFLD is a broad disease that is not related to alcohol consumption, but could potentially progress into later stages of liver diseases, such as NASH, hepatic fibrosis, and cirrhosis [57]. Additionally, some cases of NAFLD may develop into liver cancer [54]. The current spectrum of NAFLD progression with the addition of ductular reaction is proposed in Figure 4.

## 3. Role of Cholangiocytes in NAFLD

### 3.1. Cholestasis and Cholangiopathies Associated with NAFLD and NASH

While NAFLD is typically considered a disorder of hepatocytes, the underlying role of other liver cell types, including cholangiocytes, is becoming more known. Recent work has begun to unravel the impact of cholangiocytes and ductular reaction on NAFLD and NASH progression. For instance, NAFLD patients with cholestasis have more advanced histological impairments, including cholangitis, advanced fibrosis, and cirrhosis when compared with age- and sex-matched NAFLD cohorts [58]. In fact, pediatric NASH patients, and a subset of adult NASH patients, present with portal fibrosis, correlated with ductular reaction and HPC proliferation, and in adult NASH patients, portal fibrosis correlated with NASH activity, portal and centrilobular inflammation, ballooning, and NAFLD activity score (NAS) [59]. In addition, HPC expansion is associated with periportal ductular reaction, which in turn is correlated with the stage of fibrosis [27]. In a related study, it was found that centrilobular ductular reaction significantly correlated with necroinflammation and fibrosis scores in NASH patients [28]; however, it would be interesting to compare the impact of periportal versus centrilobular ductular reaction to understand if there are zonal disparities. These studies link ductular reaction and, by association, damaged ducts with NASH phenotypes.

**Figure 5:**
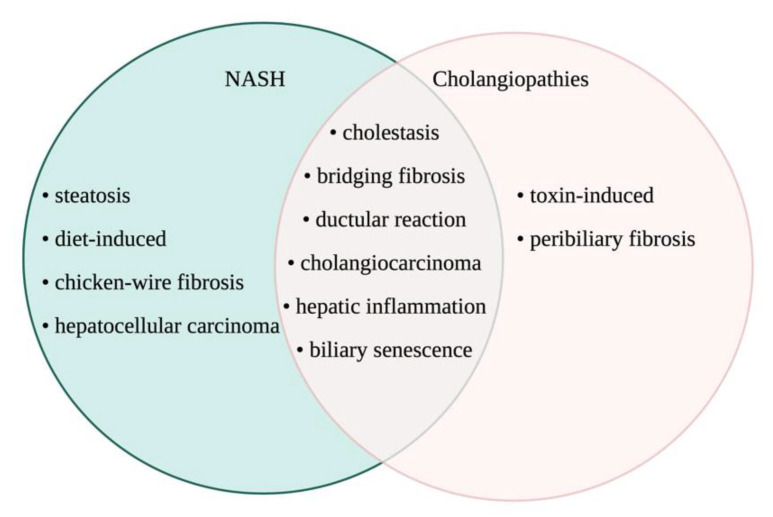
Liver phenotypes between NASH and cholangiopathies. Despite not being considered as a classic disease of the biliary epithelium, NASH shares common etiology and phenotype with cholangiopathies. This Venn diagram presents the comparative scenario on how NASH can be considered a pathological condition of the biliary epithelium owing to prominence of the most common features of cholangiopathies like PSC, PBC, and biliary atresia. Image made with BioRender (https://biorender.com/).

Considering cholangiopathies and NAFLD both impact liver function, researchers have looked into the association between these two disorders. A large multi-center study conducted by the NASH Clinical Research Network found that 5% of NASH patients in their cohort were AMA-positive without histological presentation of PBC [60], which has been supported by another study [61]. While AMA-positive NASH patients did not present with bile duct injury, it is hypothesized that detection of serum AMA may present years to decades before cholestatic or PBC manifestation [61], alluding to an interaction between bile duct damage and NAFLD in a subset of patients. An additional observational study reported an association between severe bile duct injury and fibrosis in patients with PBC who also express NASH and a BMI ≥ 25 (i.e., overweight) [62], again highlighting a close association between bile duct damage and NAFLD, specifically in the setting of PBC. Aside from PBC, NAFLD may be interlinked with other cholangiopathies. It is largely understood that NAFLD is a risk factor for the development of hepatocellular carcinoma, but one study found that NAFLD may increase the risk of CCA development [63]. This work is supported by other findings indicating that obesity may promote CCA progression, as indicated by larger tumor size and increased metastasis in obese CCA patients compared with normal weight counterparts [64]. Others have found that NASH, but not NAFLD, was a significant risk factor for CCA development and worse prognosis [65]. Interestingly, one study found that patients with PSC and IBD were less likely to develop NAFLD when compared with patients with IBD alone [66]; however, this study did not compare NAFLD in PSC (with or without IBD comorbidity) to NAFLD alone, thus more work is necessary. Overall, it is important that we understand the impact of the bile ducts and biliary-derived factors on NAFLD progression and outcomes. The overlap in known biliary and liver damages in NASH and typical cholangiopathies is described in Figure 5.

### 3.2. Ductular Reaction and Biliary Damage in NAFLD/NASH 

It is apparent that there is a link between biliary disorders and NAFLD, thus understanding changes in bile duct morphology in the setting of NAFLD may be important for identifying patient outcomes. One study found that 90.4% of cirrhotic NAFLD patients in their cohort presented with reactive bile ducts, and specifically these changes were found in the intrahepatic large septal bile ducts, with no changes noted in the small intrahepatic bile ducts [67]. Interestingly, the authors found that these reactive bile ducts were not associated with CCA presence [67]; however, this study had too small of a patient population to perform significant statistical analysis, and the cohort obtained only Indian patients, thus additional large-scale studies across different populations are necessary. Another study found that the number of bile ductules increased in NASH and correlated with portal inflammation and fibrosis [68]. These bile ductules showed increased expression of senescence markers and the SASP marker C-C motif chemokine ligand 2 (CCL2), which may be responsible for hepatic stellate cell (HSC, pro-fibrogenic liver cell) activation in NAFLD [68]. The role of biliary senescence on HSC activation and fibrogenesis is important as it is hypothesized to be a key player in cholangiopathies [69,70]. In pediatric NAFLD, activation of HPCs has also been discussed, and interestingly, these HPCs expressed adiponectin, resistin, and glucagon-like peptide-1 (GLP-1), which were associated with NAFLD severity [71]. Adiponectin, resistin, and GLP-1 are influential in metabolic disorders, thus these findings introduce the concept that biliary signaling may modulate insulin resistance and other metabolic diseases associated with NAFLD.

A recent study hypothesized that patients with NAFLD or NASH may experience cholangiocyte injury through lipoapoptosis [57]. As NAFLD patients have increased free fatty acid (FFA) concentrations, hepatocyte lipoapoptosis is a typical feature of NAFLD and is a major determinant of fibrogenesis during disease progression [57,72]. Researchers tested the saturated FFAs palmitate and stearate on cholangiocytes in vitro and found that FFAs induced cholangiocyte lipoapoptosis, similar to hepatocyte lipoapoptosis [57]. Furthermore, the researchers attempted to gain insight into whether cholangiocytes would accumulate lipid droplets similar to hepatocytes treated with FFAs, but the results indicated that, although cholangiocytes may experience lipoapoptosis after FFA treatment, they did not store lipid droplets [57]. Interestingly, one study found that 12-month-old liver-specific adipose triglyceride lipase (ATGL) knockout mice displayed severe hepatic steatosis with the presence of cytoplasmic lipid droplets in cholangiocytes [73]; however, this is a genetic model of induced liver steatosis and may not be physiologically relevant. The impact of lipid mediators on cholangiocytes is significant considering one study found that enhanced hepatocyte lipogenesis induced cholangiocyte proliferation, which in turn promoted hepatocarcinogenesis in a zebrafish model [74].

Gallstone formation is a common health issue and is likely related to NAFLD and enhanced CCA risk [75,76]. According to recent research, there appears to be a correlation between hypoxia-inducible factor 1α (HIF1α), gallstone development, and bile changes [75]. The liver is naturally a hypoxic area and conditions such as NAFLD will further promote hypoxia within the liver [75,77]. This particular study observed the significance of HIF1α in gallstone formation and biliary phenotypes within NAFLD conditions, and after 1 week of feeding a cholesterol and cholate-rich diet (CCD) in mice, 0% gallstone formation was observed in hepatocyte-specific HIF1α knockout (iH-HIFKO) mice compared with 60% gallstone formation in control mice [75]. Even after 2 weeks of feeding, there was a significant reduction in gallstone formation in iH-HIFKO mice compared with control mice [75]. Researchers also found that patients with NAFLD had increased HIF1α expression in total liver, hepatocytes, and cholangiocytes compared with the healthy subjects, and as a result, they concluded that reduced levels of HIF1α correlate with decreased gallstone formation [75]. Most importantly, this work found reduced levels of bile acids, cholesterol, and lipids in bile and [78] enhanced bile flow in CCD-fed iH-HIFKO mice compared with control mice [75]. The authors found that enhanced bile flow was due to increased hepatocyte water secretion into the bile canaliculi, and not due to cholangiocyte water secretion; however, it was noted that CCD-fed iH-HIFKO mice had reduced biliary bicarbonate output, demonstrating an interaction between hepatocytes and cholangiocytes during CCD-induced NAFLD [75]. In NAFLD patients, gallstone disease is significantly associated with advanced fibrosis and necroinflammatory activity, without changes in steatosis [78].

### 3.3. Bile Acid Signaling

Enhanced bile acid levels are noted in patients with NASH [31,79], showing that bile acid signaling may be a key determinant of NASH progression or outcomes. Indeed, mice with combined steatosis and cholestasis have worse inflammation and hepatic fibrosis than mice with cholestasis alone, which is hypothesized to be due to enhanced bile acid levels and a more cytotoxic bile acid composition [80]. These studies indicate that cholestatic injury and bile acid homeostasis can impact NAFLD injury.

A study observing obese Zucker rats at 8 and 14 weeks of age found that the obese rats in both age groups exhibited reduced bile secretion and canalicular transport capacity when compared with controls [81]. Interestingly, Mrp2 expression was significantly reduced in both the 8- and 14-week-old obese rats by an average of 70% compared with lean rats [81]. These findings are in line with another study that found mislocalization of canalicular MRP2 in humans with NASH and rodents fed a methionine and choline-deficient diet [82]. Furthermore, *ABCC2* (MRP2 gene) polymorphisms are significantly associated with NAFLD development and severity [83]. Aside from MRP2, changes in other bile acid transporters may be key in NAFLD progression and outcomes. One study found significant hypermethylation of *SLC51A* (OSTα gene) in NAFLD and NASH patients versus control [84]; however, changes in transcription and function were not noted. Ostα^−/−^ mice are resistant to age-related weight gain and hepatic steatosis [85]. Furthermore, these mice present with increased fecal lipid excretion, indicating altered intestinal lipid absorption [85]; however, this research does not underline Ost roles in cholangiocytes, and does not evaluate outcomes following Western diet or high fat diet feeding. Modulation of bile acid uptake may be important for modulation of NAFLD-associated liver damage.

In rodents, consumption of a high sucrose diet (HSD) has been shown to decrease bile formation and increase lipid retention within the liver [86]. Researchers studied NAFLD in rats with hereditary hypertriglyceridemia (HHTg) while being fed a HSD and tested the effects of boldine, an FXR agonist derived from Chilean Boldo tree, as a therapeutic substance for NAFLD treatment [86]. HSD reduced bile formation via reduced cholangiocyte bile secretion. Additionally, boldine treatment partially reduced cholestatic injury in HSD-fed mice via increased Bsep and Ntcp expression and activity [86]. Opposite to this, one study found that heterozygous knockout of Bsep reduced high fat diet-induced liver steatosis in mice [87]. However, others have shown that Ntcp deficiency protects from high fat diet-induced liver steatosis in mice [88]. In total, changes in bile acid transporters, potentially mediated by FXR, modulate NAFLD injury; however, additional work on the impact of biliary damage and ductular reaction is required.

As mentioned, FXR may contribute to the development of hepatic steatosis; therefore, treating NAFLD patients with an FXR agonist may have beneficial effects [86]. The FXR agonist INT-767 reduces liver steatosis and hepatic inflammation in rats fed a high fat diet [89]. Similar to these studies, in mice fed a high fat diet, FXR agonism is shown to induce TGR5 expression, subsequently promoting cAMP signaling and leading to improved hepatic lipid metabolism [90]. Indeed, one review summarized the potential use of TGR5 activators in the amelioration of NAFLD phenotypes via modulation of bile acid synthesis, lipid metabolism, and inflammation [91]; however, considering TGR5 is a key biliary receptor that mediates bile composition and bicarbonate secretion, it is important to look at impacts on the biliary tree. The pre-clinical work on FXR has been translated into a therapeutic option via the development of the FXR agonist, OCA. In the recent phase 3 REGENERATE study, OCA has achieved the interim histological endpoint of fibrosis improvement without worsening NASH, which is the first positive phase 3 study in NASH. This study is currently being followed for long-term clinical outcomes [92].

**Figure 6:**
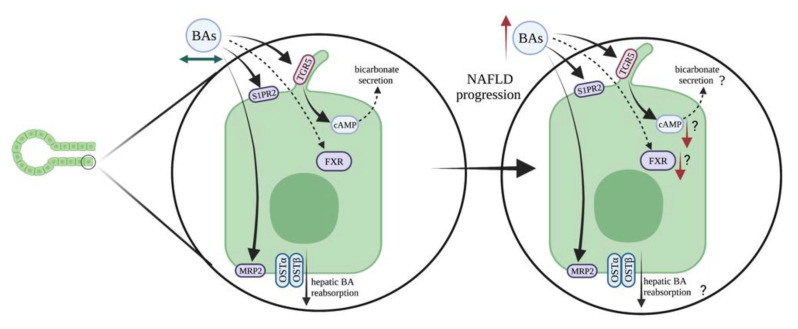
Potential roles of bile acid signaling in NAFLD progression. Under homeostatic conditions in the liver, TGR5 and S1PR2 are two of the apical membrane bile acid (BA) receptors present on cholangiocytes and are predominantly responsible for bile acid signaling across the biliary epithelium. An increase in total bile acids is a feature observed in NAFLD patients and in murine models. This can be primarily a result of reduced expression and localization of TGR5 or S1PR2 on the apical membrane and reduced FXR activation in cholangiocytes, which usually binds to bile acids and facilitates downstream pathways. This event might be considered as the initiation of a cascade of signaling events that ultimately leads to disruption of bile homeostasis across the biliary epithelium, thereby contributing to the NAFLD phenotype. Image made with BioRender (https://biorender.com/).

Conjugated bile acids activate S1PR2, which leads to downstream modulation of hepatic glucose and lipid metabolism [93,94]. One study found that 20-week-old *S1pr2^−/−^* mice developed hepatic steatosis [95]. Downstream of S1PR2 activation is the induction of sphingosine kinase 2 (SPHK2) that can translocate to the nucleus to modulate gene synthesis, and in the same study above, the authors found that *Sphk2^−/−^* mice at 20 weeks of age spontaneously develop hepatic steatosis [95]. This study did not evaluate changes in ductular reaction or cholangiocyte biology; however, other work has indicated that S1PR2 signaling promotes bile acid-induced cholangiocyte proliferation and cholestasis [43]. Another study found that blocking S1PR2 enhanced liver steatosis and HCC development in a melanocortin-4 receptor-deficient murine model of NASH [96]. Biliary S1PR2 signaling may play a role in NAFLD/NASH progression, but direct analysis is needed. Potential changes in biliary bile acid signaling during NAFLD/NASH are depicted in Figure 6.

### 3.4. Biliary Neuroendocrine Phenotype

#### 3.4.1. Vascular Endothelial Growth Factor (VEGF)

As indicated above, significant alterations of the biliary tree and ductular reaction are noted in NAFLD and NASH, and may be associated with disease severity. Injured cholangiocytes have been identified as a neuroendocrine compartment through their release of various cytokines, growth factors, neuropeptides, and hormones that can regulate disease progression [46]. VEGF is released from neuroendocrine-like cholangiocytes, and has proliferative and angiogenic roles in NAFLD [97]. So far, one study has found that VEGF and angiopoietin 2 (Ang2) were significantly increased in NASH patients and correlated with ductular reaction [98]. While descriptive, this study alludes to the role of VEGF in ductular reaction during disease progression. This is the only article linking angiogenic signaling with biliary dysfunction in NAFLD/NASH; however, a close association between the hepatic vasculature, angiogenic factor signaling, and cholangiocytes is known during cholestatic injury and warrants further investigation into NAFLD/NASH [99].

#### 3.4.2. Cannabinoid Signaling

Neuroendocrine-like cholangiocytes can express the cannabinoid receptor 2 (CB2). It was found that both cholangiocytes and hepatocytes express cannabinoid receptor 2 (CB2) in NAFLD patients [100]. Interestingly, cholangiocytes stained positive for CB2 during steatosis or NASH, but were negative for this receptor in control samples [100]. Interestingly, CB1 and CB2 expression is found in cholangiocytes in patients with PBC when compared with controls [101]. In a previous study, the activation of CB2 receptor has been shown to have antifibrogenic properties and to be stimulated during cirrhosis [102]. However, the function of CB2 in NAFLD and cholangiopathies is still poorly defined.

#### 3.4.3. Histamine/Histamine Receptor (HR) Axis and Mast Cells (MCs)

Patients using H2HR antagonists have a lower prevalence of NAFLD [103]. As previously stated, inhibition of histamine signaling and H1 and H2 HR activation reduces biliary damage and liver fibrosis in models of cholestasis; therefore, there may be a link between biliary-derived histamine and NAFLD progression. One study found that l-histidine decarboxylase (Hdc) knockout (*Hdc^−/−^*) mice were protected from HFD-induced ductular reaction, biliary senescence and subsequent liver fibrosis [104]. Interestingly, these mice developed more steatosis when challenged with HFD owing to the loss of histamine/leptin signaling, leading to leptin resistance [104]. This paper alludes to the role of autocrine biliary signaling; however, it has been hypothesized that hepatic mast cells (MCs) may be a key contributor to hepatic histamine levels in cholestasis. Patients with PSC and CCA have increased MC numbers, which are found in close proximity to bile ducts [105], and loss or inhibition of MCs reduces biliary damage and liver fibrosis associated with cholestatic mouse models [105]. A recent study found that both patients with NAFLD/NASH and mice fed Western diet (WD) had increased numbers of MCs, which were found surrounding bile ducts [105]. The authors next utilized a model of MC-deficiency, *Kit^W-sh^* mice, subjected to WD feeding, and found that there was a marked amelioration of NAFLD phenotypes including ductular reaction, inflammation, and hepatic fibrosis when compared with wild-type (WT) mice fed WD [105]. Of note, the authors found that MCs were able to predominantly alter microvesicular steatosis, which is the steatotic phenotype predominantly found in NASH and indicating a worse prognosis [105]. To verify their findings, the authors demonstrated that re-introduction of MCs into the MC-deficient or WT mice fed WD induced greater damage compared with mice fed WD alone.

#### 3.4.4. Toll-like Receptor 4 (TLR4)

Damaged cholangiocytes upregulate their expression of toll-like receptors (TLRs) to induce pro-inflammatory responses, and one study found that the expression of TLR4, which regulates host response to pathogens, was upregulated in patients with NAFLD [106]. Of interest, the authors found that TLR4 expression was highly expressed in HPCs, biliary cells, and portal/septal macrophages [106]. The degree of TL4-positive HPCs and bile ducts significantly correlated with inflammation and fibrosis in NAFLD patients as well [106]. Researchers also found a significant relationship with the degree of liver disease and the amount of activated TLR4 HPCs [106]. These findings are significant considering that TLR4 activation has been linked to biliary protection in relation to biliary innate immunity [99].

#### 3.4.5. Osteopontin (OPN)

Osteopontin (OPN) is typically expressed by bile ducts and can regulate cell proliferation, inflammation, and fibrogenesis, but the impact on NAFLD progression is unknown. One study found that OPN expression was enhanced in NASH patients, specifically in the bile ducts [107]. Interestingly, enhanced biliary OPN expression correlated with fibrogenesis in NASH patients [107]. These findings were recapitulated in a mouse NASH model, and the authors further found that loss of OPN reduced liver fibrosis in their model [107]. Furthermore, cholangiocyte-derived OPN promoted HSC activation and fibrogenesis [107]. Interestingly, the authors linked enhanced biliary OPN expression to increased hedgehog (Hh) signaling, which was verified in mice with overly active Hh signaling that demonstrated worsened NASH-associated liver fibrosis [107]. Hh signaling has been linked to ductular reaction and other pathological responses in cholangiocytes during cholestasis [108], thus this pathway may be important for ductular reaction-driven NAFLD responses.

#### 3.4.6. Secretin/SR Signaling and Associated CFTR Activation

Secretin/SR signaling is key for biliary secretory responses during cholestasis, but the impact on NAFLD is undefined. Two different studies analyzed outcomes in *Sr*^−/−^ mice subjected to high fat diet feeding [26,109]. One study found that *Sr*^−/−^ mice were resistant to high fat diet-induced weight gain, and attributed these outcomes to impaired intestinal lipid absorption in these mice [109]. A separate study evaluated the impact of *Sct*^−/−^ and *Sr*^−/−^ in mice fed a high fat diet, and the authors found that loss of this signaling axis reduced liver steatosis, ductular reaction, inflammation, and fibrosis in their NAFLD model [26]. One of the interesting aspects of this study was the impact of Sct/SR signaling (only found in cholangiocytes in the liver) on hepatocyte lipogenesis. The authors further delved into this mechanism and found that Sct/SR-dependent downregulation of miR-125b is able to promote steatosis via enhanced elongation of very long chain fatty acids’ protein 1 (Elovl1) expression [26]. Importantly, similar changes in the SCT/SR/miR-125b/ELOVL1 axis were found in NAFLD patients [26]. Interestingly, the results indicated that WT mice gained significantly more weight and exhibited a higher body fat percentage than *Sr^−/−^* mice [26,109]. Another key finding was that *Sct*^−/−^ and *Sr*^−/−^ mice subjected to a high fat diet had enhanced bile acid excretion and significant alterations of bile acids metabolized by the gut microbiota [26]. 

**Figure 7:**
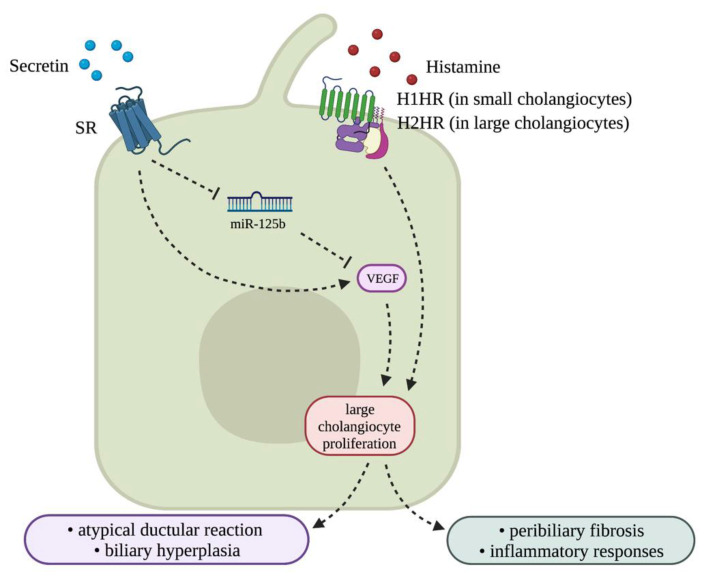
Key neuroendocrine signaling that may play a role in NAFLD. The neurohormones involved in cholestasis are mainly secretin and histamine. Through signaling via secretin receptor (SR) and histamine receptors (H1HR and H2HR), respectively, cholangiocytes can assume a neuroendocrine phenotype during cholestasis that further leads to atypical ductular reaction, biliary hyperplasia, and inflammation. In NAFLD, it has been shown indirectly that elevation of secretin and histamine is a key phenotype, thus suggestive of the role of these neurohormones in the progression of NAFLD. Image made with BioRender (https://biorender.com/).

Downstream of Sct/ SR signaling is activation of CFTR, which mediates bicarbonate-rich choleresis. A unique case study involving a pediatric cystic fibrosis (CF) patient found diffuse fatty liver that gradually developed into liver cirrhosis [110]. Another study found that 14% of patients with CF-associated liver disease (CFLD) presented with liver steatosis [111]. Furthermore, to CFLD patients with steatosis tend to have higher BMIs (body mass indexes) that are similar to NAFLD patients [111]. While these studies do not specifically delve into the impact of biliary CFTR on NAFLD outcomes, there is an association that may be worth evaluating. Changes in biliary activation and neuroendocrine phenotypes during NAFLD progression are demonstrated in Figure 7.

## 4. Conclusions/Future Perspectives

This review highlights the potential impact of ductular reaction and cholangiocyte-derived factors on the promotion of disease phenotypes in NAFLD. The biliary compartment has becoming increasingly recognized for its paracrine signaling in chronic liver diseases in the last 30 years, with new investigations of this cell type in NAFLD currently on the rise. Many of the above studies found ductular reaction and biliary hyperplasia at the NASH stage, but one study found that steatotic patients present with increased bile duct mass. Indeed, considering changes in cholangiocytes early in disease could be important for prognosis, and these cells should be considered when evaluating disease progression. Most studies identify correlative findings for ductular reaction and biliary proliferation with worsening outcomes in NAFLD patients, and unfortunately causative data directly linking these findings are scarce. Some basic science studies have identified cholangiocyte-derived factors as key mediators of hepatocyte steatosis, HSC activation and subsequent liver fibrosis, but these publications are few. An extensive amount of work on damaged cholangiocyte mediation of inflammatory, immune, and fibrogenic responses for surrounding cells via paracrine signaling during cholestasis has been discovered, thus work on the direct role of cholangiocytes in NAFLD is due. Understanding the specific mechanisms modulating ductular reaction could highlight new therapeutic avenues, specifically for NAFLD patients with cholestasis that have worsening outcomes. Indeed, modulation of bile acid signaling, such as S1PR2 inhibition, could impact NAFLD outcomes. The work above highlights the dysregulation of bile acid signaling and synthesis during NAFLD, and as cholangiocytes can secrete and modify bile acids, targeting this cell source may prove important. Furthermore, inhibition of secretin or histamine signaling could prove therapeutic for NAFLD patients. Targeting these two signaling pathways has been extensively shown in cholestatic models, thus their translation to NAFLD is unsurprising. Furthermore, HR antagonists can currently be purchased over-the-counter for general allergies and inflammation, so finding a new use for these drugs could be beneficial. Owing to the lack of treatment options available, continued research related to fatty liver disease and drug development is critical for NAFLD patients, and cholangiocytes may be a central regulator and target for future therapeutic interventions.
